# Diabetic Retinopathy Screening in Patients with Diabetes Using a Handheld Fundus Camera: The Experience from the South-Eastern Region in Hungary

**DOI:** 10.1155/2021/6646645

**Published:** 2021-02-08

**Authors:** Dóra Júlia Eszes, Dóra Júlia Szabó, Greg Russell, Csaba Lengyel, Tamás Várkonyi, Edit Paulik, László Nagymajtényi, Andrea Facskó, Goran Petrovski, Beáta Éva Petrovski

**Affiliations:** ^1^Department of Public Health, Faculty of Medicine, University of Szeged, Szeged, Hungary; ^2^Department of Ophthalmology, Szent-Györgyi Albert Clinical Center, Faculty of Medicine, University of Szeged, Szeged, Hungary; ^3^Eyenuk Inc., Clinical Development, Woodland Hills, CA, USA; ^4^Department of Medicine, Medical Faculty, Albert Szent-Györgyi Clinical Center, University of Szeged, Szeged, Hungary; ^5^Center for Eye Research, Department of Ophthalmology, Oslo University Hospital and Institute for Clinical Medicine, Faculty of Medicine, University of Oslo, Oslo, Norway; ^6^The A. I. Evdokimov Moscow State University of Medicine and Dentistry of the Ministry of Healthcare the Russian Federation, Moscow, Russia

## Abstract

**Purpose:**

Diabetic retinopathy (DR) is the leading cause of vision loss among active adults in industrialized countries. We aimed to investigate the prevalence of diabetes mellitus (DM), DR and its different grades, in patients with DM in the Csongrád County, South-Eastern region, Hungary. Furthermore, we aimed to detect the risk factors for developing DR and the diabetology/ophthalmology screening patterns and frequencies, as well as the effect of socioeconomic status- (SES-) related factors on the health and behavior of DM patients.

**Methods:**

A cross-sectional study was conducted on adults (>18 years) involving handheld fundus camera screening (Smartscope Pro Optomed, Finland) and image assessment using the Spectra DR software (Health Intelligence, England). Self-completed questionnaires on self-perceived health status (SPHS) and health behavior, as well as visual acuity, HbA1c level, type of DM, and attendance at healthcare services were also recorded.

**Results:**

787 participants with fundus camera images and full self-administered questionnaires were included in the study; 46.2% of the images were unassessable. T1D and T2D were present in 13.5% and 86.5% of the participants, respectively. Among the T1D and T2D patients, 25.0% and 33.5% had DR, respectively. The SES showed significant proportion differences in the T1D group. Lower education was associated with a lower DR rate compared to non-DR (7.7% vs. 40.5%), while bad/very bad perceived financial status was associated with significantly higher DR proportion compared to non-DR (63.6% vs. 22.2%). Neither the SPHS nor the health behavior showed a significant relationship with the disease for both DM groups. Mild nonproliferative retinopathy without maculopathy (R1M0) was detected in 6% and 23% of the T1D and T2D patients having DR, respectively; R1 with maculopathy (R1M1) was present in 82% and 66% of the T1D and T2D groups, respectively. Both moderate nonproliferative retinopathy with maculopathy (R2M1) and active proliferative retinopathy with maculopathy (R3M1) were detected in 6% and 7% of the T1D and T2D patients having DR, respectively. The level of HbA1c affected the attendance at the diabetology screening (HbA1c > 7% associated with >50% of all quarter-yearly attendance in DM patients, and with 10% of the diabetology screening nonattendance).

**Conclusion:**

The prevalence of DM and DR in the studied population in Hungary followed the country trend, with a slightly higher sight-threatening DR than the previously reported national average. SES appears to affect the DR rate, in particular, for T1D. Although DR screening using handheld cameras seems to be simple and dynamic, much training and experience, as well as overcoming the issue of decreased optic clarity is needed to achieve a proper level of image assessability, and in particular, for use in future telemedicine or artificial intelligence screening programs.

## 1. Introduction

Diabetes mellitus (DM) is a major medical and societal challenge due to its rapid increase in global prevalence and devastating late complications [1, 2]. The global occurrence of DM among adults (>18 years of age) was 8.5% in 2014, and this has nearly doubled from its 4.7% level in 1980 [3]. In 2016, 1.6 million deaths were directly attributed to DM, with more than half of them occurring in the lower- and middle-income countries. According to the WHO forecast, DM will be the seventh leading cause of death in 2030, while diabetic retinopathy (DR) will be the leading cause of vision loss among active adults in industrialized countries [4]. DR is the most common late complication of DM in people aged 20 to 64 years—the working-age population, and except for where effective screening programs have been implemented, it is the leading cause of blindness and reduced vision in this group in the developed world [5, 6]. In a study comparing data from 35 populations, the global prevalence of sight-threatening retinopathy (STR) was estimated at 10.2% for all DM patients [6].

In Hungary, a total of 865 069 patients (9.5% of the population) suffered from DM among adults (>18 years of age) in 2011 [7], and some degree of DR could be observed among 19% of the patients with type 1 DM (T1D) and 24% in those suffering from type 2 DM (T2D) for 3 or 4 years [8]. Systematic DR screening and monitoring has been proven to be cost-effective in reducing blindness and visual impairment in patients having DM. Screening enables optimized timing of laser and medical therapy that may halt disease progression [9]. The WHO guidelines [10] for DR screening state that “annual eye examinations are recommended for patients with diabetes (and every other year for persons with excellent glycemic control and no retinopathy at the previous examination...).” “Such programs need systematic evaluation for their impact on health outcomes, cost effectiveness and health equity.” The WHO recommendation further states “Member States should choose the most appropriate interval between examinations” [10].

The development of optimized and effective DR screening programs is becoming eminent. The aim of this study was to investigate the prevalence of DR and its different grades in patients with DM in the Csongrád County—a South-Eastern region in Hungary, using for the first time in this country a handheld fundus camera (Smartscope Pro Optomed, Finland). Moreover, we aimed to detect the risk factors for developing DR and the diabetology/ophthalmology screening patterns and frequencies, as well as the effect of socioeconomic status- (SES-) related factors on the health and behavior of DM patients.

## 2. Patients and Methods

### 2.1. Physical Examination

A cross-sectional study was conducted between the Departments of Ophthalmology and Internal Medicine Diabetology Unit, University of Szeged, Szeged, Hungary, between November 2015 and December 2016. All examinations were voluntary and free of charge to the participants, and the patients were recruited consecutively from the Diabetology Outpatient Clinic. Written informed consent was obtained from all participants. The study was approved by the local ethical committee of the University of Szeged (No.197/2015). The detection of DR was based upon examination with a handheld fundus camera (Smartscope Pro Optomed, Finland) in a dark room by qualified professionals. The results were directly evaluated by a qualified specialist without the need to do data/file transfer. In the case of constricted pupil, another image was taken after ensuring normal intraocular pressure level and applying cyclopentolate (5 mg/mL) eye drops to achieve mydriasis. The assessment of the fundus images was performed using the Spectra DR software (Health Intelligence, UK). The recordings were safely deposited and kept inaccessible to third parties for 10 years at a designated server, so that later they can be used in further comparative studies on DR.

The images acquired with the Optomed Smartscope Pro digital handheld camera included two pictures from the participants' eyes—one with the macula—and another with the optic nerve—in the center—which is in line with the English screening requirements [11]. In case of presence of amblyopia or nontransparent media (e.g., cataract and corneal or visual axis obstructing conditions), the patients were excluded from the study. During image evaluation, the graders (A.F./G.P./G.R.) classified the signs and stages of DR and maculopathy in the standardized English-based software Spectra DR and graded the images in alignment with the English standard grading protocols [12]. Each image was evaluated in two stages: first, the referral outcome graders/ROGs (D.E./G.R.) evaluated them, and then a supervisor/ophthalmic consultant confirmed the diagnosis (A.F./G.P.). At the end, an expert opinion regarding the grade of retinopathy was provided, which included the stage of retinopathy (R0/1/2/3A) and the absence or existence of maculopathy (M0/1). Other discovered abnormalities were not diagnosed in this study, although they were recorded, as they can provide further information about other symptoms, which may have occurred in the past, and therefore may require medical attention over a specified period of time.

The classification of the DR has been described before [13]—in brief: (R0) no clinical anomaly—repeated screening was recommended one year later; (R1) mild nonproliferative—presence of microaneurysms, dot- or blot- like hemorrhages, or exudates—control examination was recommended one year later; (R2) moderate or severe nonproliferative—presence of major bleeding(s) and intraretinal microvascular abnormalities (IRMAs)—control examination was required within one month; (R3A) active proliferative—presence of neovascularization of the optic disc (NVD) or elsewhere (NVE) or preretinal bleeding(s), vitreous bleeding, preretinal fibrosis, and tractional retinal detachment—immediate medical examination was required within two weeks. All the stages were combined with sight-threatening maculopathy which was determined by the presence of exudates regardless of visual acuity (VA), or red lesions with a VA of 6/12 or worse after pinhole correction, that is within 1 disc diameter of the center of the fovea, and/or a group of exudates where the area of exudates that is greater than or equal to half the disc area, and this area is all within the macular area (as defined by the ETDRS macular grid) when medical examination was required within a month (M1).

### 2.2. Self-Completed Questionnaire

Participants were asked to fill out a self-administered questionnaire which was based upon the European Health Interview Survey 2009—it included demographic characteristics such as gender, age, and place of residency. From the place of residency, the distance to the healthcare facility was calculated as <10 km or ≥10 km.

The marital status was categorized as married or lives with a partner, single, separated or divorced and widowed; due to the low sample size, categories were merged together as living alone or living in partnership. SES of the study participants was examined: education and economic status. The economic status was characterized as working—full time and working—part-time, unemployed, retired, temporarily laid off, and student; due to the lack of data between each category, the categories were allocated and merged as inactive or active. The level of education was measured as primary, secondary, or higher education (college, university, or higher).

Data were collected about self-perceived health status (SPHS) and characterized as bad satisfactory, and good. Information was also collected about “Perception of what the subject can do for his/her health status,” and the information was categorized as almost nothing (nothing/little) or much more (much/very much).

Health behavior was assessed by alcohol consumption, smoking, physical activity, and diet (no/yes). Smoking was classified as yes/quit/never smoking, while alcohol consumption was classified as no/yes. Physical activity was defined according to the amount or occasions spent in the previous month in cycling, walking: daily/weekly more time, weekly, once/no activity at all (inactive).

Information was also collected about the DM-related and other health conditions, for example, if the study participant has/had hypertension: no/yes. If yes, data were collected about the duration of the hypertension (years). If the participant attended blood pressure controls, a recording was made about the last measurement of the systolic and diastolic blood pressures in millimeters of mercury (mmHg).

Information was further collected about other health conditions, for example, VA (<0.3 or ≥0.3), HbA1c level (normal <7% or elevated ≥7%), type of diabetes mellitus (T1DM or T2DM), use of medications, DM in the family or occurrence of diabetic maculopathy. In addition, data about the attendance at healthcare services like diabetology (monthly, every 3^rd^ month, every 6^th^ month, yearly, more than a year, or no attendance) were also collected.

### 2.3. Statistical Analysis

The analysis of the data was performed by descriptive statistics; percentage distribution, mean and standard deviation (SD), and in case of nonnormality of continuous variables, median and interquartile range (IQR) and range (minimum, maximum) are shown. Normality of the continuous variables was tested on a histogram, Q-Q- plot, and by Shapiro-Wilk and Kolmogorov-Smirnov test. The Independent Sample *T*-test was used to compare the means of the continuous, numerical variables, when the normality assumption was satisfied; otherwise, Mann–Whitney *U* test was used. Homogeneity of variance was analyzed with the Levene test.

Chi-square (*χ*^2^) and Fisher test were used to test the differences of the distribution of categorical variables; for multiple comparisons, the 2-sample *z*-test with Bonferroni correction was applied to detect the differences in the proportions between the studied groups. If the sample within each column was 1 or less, then the *z*-test could not be used. The significance limit was set at *P* < 0.05. The statistical analysis of the data was performed by IBM SPSS Statistics Version 24 software.

### 2.4. Ethical Issues

The Regional and Institutional Human Medical Biological Research Ethics Committee of the Szent-Györgyi Albert Clinical Center, University of Szeged approved the study protocol (No. 197/2015). The research provided anonymity to the participants. Before the beginning of a test, the participants signed a voluntary written consent form in which they agreed to permit the use of data for research purposes.

## 3. Results

**Figure 1:**
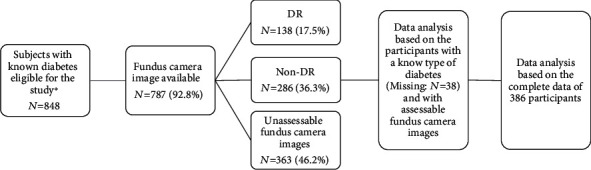
Flowchart of the study sample. DR: diabetic retinopathy; Non-DR: nondiabetic retinopathy; N: number. ^∗^Fulfilled the self-completed questionnaire and had a fundus camera image taken.

The data were collected from a total of 848 participants with known DM in the Csongrád County, South-Eastern region in Hungary (Figure 1). Out of the initial participants, 787 (92.8%) had available fundus camera images and answered the self-administered questionnaire. T1D was present in 13.5% (*N* = 52) of participants, while T2D was present in 86.5% (*N* = 334) of the participants. Among the T1D and T2D patients, 25.0% (*N* = 13) and 33.5% (*N* = 112) had DR, respectively. A large portion of the participants had unassessable fundus camera images/results 46.2% (*N* = 363) when using the handheld camera, and therefore excluded from the further analysis (Figure 1).

The data analysis was based upon the remaining 386 individuals, who had assessable fundus camera images and possessed complete data about the type of diabetes and the risk parameters studied.

Table 1 shows the characteristics of the studied participants. Gender, age, and marital status showed no significant proportion differences between the study groups, while SES showed significant proportion differences in the T1D group. The proportion of the DR differed significantly in the Education and Perceived Financial Status groups, and it was significantly higher among those with higher education (secondary/higher being 61.5%/30.8%) and perceived bad financial status (63.6%). The distance travelled to the healthcare service showed a nearly significant association with the DR—participants living more than 10 km away from the healthcare services had a higher proportion of DR (61.5%).

Tables 2 and 3 show the results of the SPHS and the health behavior of the individuals, neither of which showed a significant relationship with the disease for both, T1D and T2D groups.

Table 4 shows the characteristics of the health status of the study participants. A significant difference was only present in case of diabetes medication use and presence of diabetic maculopathy in T2D patients having DR and non-DR, with the rest of the parameters included (hypertension, VA, HbA1c, duration of DM, and familiar presence of DM) showing no significant proportion differences between the studied groups.

**Figure 2:**
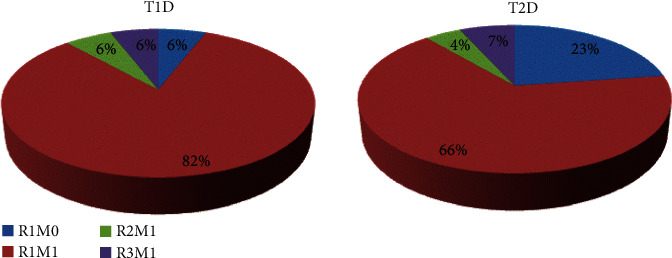
Distribution of the diabetic retinopathy according to the type of diabetes mellitus. DM: diabetes mellitus; T1D and T2D: type 1 and 2 DM.

Mild nonproliferative retinopathy without maculopathy (R1M0) was detected in 6% of the T1D patients having DR, and 23% of the T2D patients having DR. Among the patients having DR, R1 with maculopathy (R1M1) was present in 82% of the T1D group, and 66% of the T2D group. Both moderate nonproliferative retinopathy with maculopathy (R2M1) and active proliferative retinopathy with maculopathy (R3M1) were detected in 6% of the T1D patients having DR. Among the T2D patients having DR, the prevalence of R2M1 was 4%, while the prevalence of R3M1 was 7% (Figure 2).

**Figure 3:**
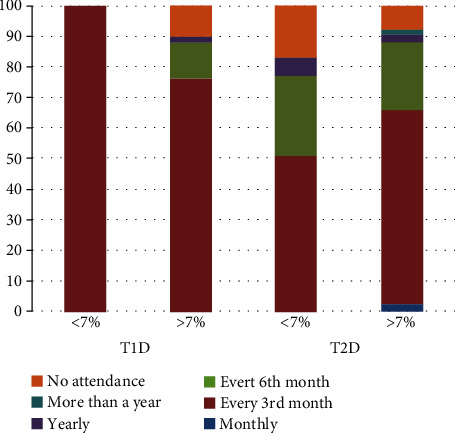
Attendance rate in the diabetology screening among those with normal or elevated HbA1c. T1D: type 1 diabetes mellitus; T2D: type 2 diabetes mellitus. ^∗^Data presented are based upon the result of 1 individual in case of the T1D group having HbA1c <7%.

The level of HbA1c affected the participation in the diabetology screening, with those having HbA1c > 7% representing more than 50% of all quarter yearly attendance for both types of DM (Figure 3). About 10% of the population had no diabetology screening attendance for those having HbA1c > 7% for both types of DM and HbAc < 7% T2D. For both types of DM, the yearly attendance was below 5%, while more than yearly attendance was absent for all studied groups, and low for T2D patients having HbA1c > 7% (Figure 3).

## 4. Discussion

DR is the most common late complication of DM in the working-age population and the leading cause of blindness in the elderly, accounting for a significant drop in the quality of life (QoL) and working ability for the patients [5, 14]. In a study comparing data from 35 populations, the global prevalence of sight-threatening retinopathy (STDR) was estimated to 10.2% for all DM patients [6]. Our study found high rates of R2M1 and R3M1, moderate and active proliferative retinopathy (6% and 7% for T1D and T2D, respectively), which is similar to the world average found so far.

A previous study in Hungary found the prevalence rate of DM in participants aged 20-69 years to be 7.47% [15]. More recently, a study from Hungary showed 24.5% of all incident DM cases to be T2D [16]. The same study also showed T1D to be the most common form of DM in children and adolescents, with its frequency having a tendency of continuous rising, while the occurrence of medically treated cases of T2D not to be increasing. The prevalence of T2D, however, is increasing due to an obesity epidemic and aging of the population, hence, one may expect a dramatic increase in DM during the next decades [1, 2, 10]. In the Csongrád County, South-Eastern region of Hungary, the studied cohort showed an approximate 1 : 7 ratio of T1D : T2D cases.

The population in the Csongrád County in Hungary is characterized by significant SES differences, and these appear to reflect upon significant proportion differences, in particular, in the T1D population. It has been previously reported that poorer populations having Medicaid insurance in the U.S. are associated with worse DR follow-up in predominantly rural patients [17]; this population appears to be similar to the rural population in the Csongrád County, Hungary. A statistically significant relationship between diabetes complications, age group, educational level, job status, relationship with family members, number of family visits, and the reassurance provided by the family, type of leisure activities, health status, years with diabetes, smoking, type of treatment, fried food consumption and income, sense of security and communication in living environment, and daily intake of vegetables, has also been reported in a study cohort of T2D patients [18]. Furthermore, no statistical interaction could be found between SPHS and gender, while reporting the self-perceived health as poor has been associated with higher reporting of chronic diseases, including diabetes [19].

Although hypertension, VA, HbA1c, duration of DM, and familiar presence of DM showed no significant difference in our study, another study on a population having T2D found a statistically significant difference between SPHS and the levels of HbA1c; the latter study also showed age, level of education, mode of treatment, adherence to treatment, and level of exercise to be factors having statistically significant differences from, and therefore an influence on, self-reported health in a single province in Turkey [20]. Patients with T1D have been shown to have a faster decrease in the perceived health and functioning over time compared to aged persons from the general population [21].

The distribution of the DR showed similar retinopathy with maculopathy (R1M1) presence (82% in the T1D group and 66% in the T2D group) compared to an English study on both DR types (89% had a diagnosis of R1M1 in one eye in those screened positive for maculopathy (M1) in at least one eye) [22]. Our handheld camera produced unassessable fundus image results in nearly half of the participants when used by newly trained image acquisition staff (DJE and DJS); however, in an older population having T2D, this can also be due to the presence of optic axis opacities such as cataract and vitreous hemorrhage. In our study, 6% and 7% of the T1D and T2D population, respectively, had R3M1 (proliferative diabetic retinopathy with maculopathy), while 6% and 4% of the T1D and T2D population, respectively, had R2M1 (preproliferative diabetic retinopathy with maculopathy); therefore, a total of 23% of the population had higher chance for DM-associated cataracts and or vitreous hemorrhages, as well as poor fixation due to macular edema. A limitation of our study is the fact that such changes were not recorded at the time the screening was conducted. Other studies have, however, shown that such handheld cameras can provide comparable results to standard fundus cameras [23]. Later versions of this camera (The Optomed Aurora) appear to have a built-in instant quality feedback software that aids the photographer to gain information when the image is assessable. In the latter study, the two cameras used reached high agreement on the diagnosis of retinopathy and maculopathy at all the levels of retinopathy. Sufficient training of paraprofessional health care staff can lead to obtaining higher quality images with a portable nonmydriatic fundus camera [24]. Known risk factors for developing DR are age, gender, duration and type of DM, elevated HbA_1_c, high blood pressure, and retinopathy stage, while other risk factors are being investigated. DR is caused by damage to the retinal microvasculature. Proper screening for DR is an important milestone towards achieving early and efficient treatment for preventing visual loss [9]. For optimal effect, laser treatment must be applied as early as possible after the formation of new pathological retinal vessels, at which time most patients are asymptomatic. In addition, antivascular endothelial growth factor (VEGF) drugs or steroids injected into the vitreous of the eye may reduce diabetic macular edema [25, 26]. Other European countries like Iceland, Denmark, Sweden, and England have successfully implemented nationwide DR screening programs. In Iceland, diabetic blindness prevalence has decreased 4-5 fold after the introduction of systematic DR screening, and a similar success rate has been observed in Denmark [27].

Hungary, at present, has no coordinated national screening program for DR, despite the clear need and high number of patients with DM. Furthermore, in many parts of the country, there are no clear communication channels between GPs, diabetologists, and ophthalmologists regarding screening and sharing results from a DR assessment. Today, a newly diagnosed DM patient must be actively referred for an eye examination by his/her GP or endocrinologist, and often the patient her-/himself must book the appointment. In addition, the interval between eye examinations is at the ophthalmologist's discretion. A standardized rapid assessment of avoidable blindness (RAAB) with the DR module (DRM) has recently been used in Hungary in people aged 50 years and older: 20.0% of the 3523 participants had a known or newly diagnosed DM; 20% of the participants with known DM had a blood glucose level of ≥200 mg/dL; and 27.4% had never had an ophthalmological examination for DR. The prevalence of DR and/or maculopathy was found to be 20.7%, while the prevalence of STDR was 4.3% in one or both eyes among the participants with DM in Hungary [28]. This finding is lower than the one determined in the Csongrád County in Hungary, which can certainly underline disparities in the DR grading standards used or the distributional difference of DR throughout the different counties in the country.

A systematic DR screening in the Csongrád County, South-Eastern region in Hungary, could have significantly reduced the total load of ophthalmologist exams, and thus increase the overall capacity in ophthalmology—a field with vast capacity challenges [19]. More importantly, the lack of systematic DR screening also puts patients with a high risk of eye disease progression at an even higher risk, as they are not receiving the regular follow-up examinations needed. The WHO guidelines for DR screening [5, 14] recommend annual eye examinations for patients with diabetes and biennially for persons with excellent glycemic control and no retinopathy at the previous examination. The International Council for Ophthalmology (ICO) now recommends biennial screening for DM patients without retinopathy. In general, there is a low annual incidence of STR, and 97% of the screening visits do not lead to any active treatment [29]. However, with the increasing prevalence of DM, especially T2D, and limited eye care capacity, advocating for a personalized health care approach towards patient-tailored screening and recommendation for each individual patient has been proposed.

In Iceland for example, a path of improving cost-efficacy of screening systems has been chosen by reducing the number of unnecessary screening visits. Based on a biennial screening model, the following risk variables have been included to improve risk predictions for each individual patient: age, gender, diabetes duration, type of diabetes, HbA_1_c level, blood pressure, and retinopathy stage. An European collaborative network has used this model to calculate the most appropriate interval between examinations for each patient, the outcome of which was a reduction of 17-23% in the screening visits needed, compared to the biennial screening model [29, 30]. A personalized screening approach would have the advantage of recommending more frequent screening intervals to high-risk patients and less frequent to low-risk patients. The risk variable profile also shows significant alterations between different countries and also between different ethnic- and socioeconomic populations within the same country and region, thus, the one-size-fits-all approach may not be the best for diverse populations globally.

In conclusion, this study in the Csongrád County, South-Eastern region, Hungary, determined the prevalence of DM and DR, which appeared to follow the country trend, except for the slightly higher STDR. SES appears to affect the DR rate, in particular, for T1D. The DR screening using the Smartscope Pro Optomed handheld camera, although simple and dynamic, requires much training and experience to achieve proper levels of image assessability if future use in telemedicine or artificial intelligence screening programs or personalized medicine is planned.

## Figures and Tables

**Table 1:** Characteristics of the study sample.

	T1D *N* = 52 (%)		T2D *N* = 334 (%)	
	DR *N* = 13 (%)	Non-DR *N* = 39 (%)	DR *N* = 112 (%)	Non-DR *N* = 222 (%)
Gender				
Male	7 (53.8)	23 (59.0)	47 (42.0)	94 (42.3)
Female	6 (46.2)	16 (41.0)	65 (58.0)	128 (57.7)
Age (mean ± SD)	70.8 ± 6.0	66.4 ± 12.2	66.4 ± 12.8	65.7 ± 13.0
Distance to the healthcare services				
<10 km	*5 (38.5)*	*27 (69.2)*	75 (67.0)	140 (63.4)
≥10 km	*8 (61.5)*	*12 (30.8)*	37 (33.0)	81 (36.6)^a^
Education				
Primary	*1 (7.7)*	*15 (40.5)*	54 (48.2)	94 (43.5)
Secondary	*8 (61.5)*	*10 (11.2)*	30 (26.8)	79 (36.6)
Higher	*4 (30.8)*	*12 (32.4)*	28 (25.0)	43 (19.9)^b^
Perceived financial status				
Bad	*7 (63.6)*	*8 (22.2)*	24 (23.1)	58 (27.6)
Satisfactory	*2 (18.2)*	*23 (63.9)*	70 (67.3)	131 (62.4)
Good	*2 (18.2)*	*5 (13.9)*	10 (9.6)	21 (10.0)^c^
Marital status				
Living alone	1 (7.7)	5 (13.9)	37 (33.0)	60 (27.8)
Living in partnership	12 (92.3)	31 (86.1)	75 (67.0)	156 (72.2)^d^
Economic status				
Active	9 (69.2)	21 (55.3)	*21 (18.7)*	*63 (28.9)*

*P* < 0.05. T1D: type 1 diabetes mellitus; T2D: type 2 diabetes mellitus; DR: diabetic retinopathy; Non-DR: nondiabetic retinopathy; *N*: number; SD: standard deviation. Missing data: (a) 1; (b) 8; (c) 25; (d) 7.

**Table 2:** Self-perceived health status of the study sample.

	T1D *N* = 52		T2D *N* = 334	
	DR *N* = 13 (%)	Non-DR *N* = 39 (%)	DR *N* = 112 (%)	Non-DR *N* = 222 (%)
Self -perceived health				
Bad	2 (15.4)	7 (18.4)	28 (25.2)	65 (29.3)
Satisfactory	7 (53.8)	24 (63.2)	64 (57.7)	135 (60.8)
Good	4 (30.8)	7 (18.4)	19 (17.1)	22 (9.9)^a^
What the person can do for his/her health				
Very much/much	10 (83.3)	30 (78.9)	91 (82.0)	167 (76.6)
Little/nothing	2 (16.7)	8 (21.1)	20 (18.0)	51 (23.4)^b^

*P* < 0.05. T1D: type 1 diabetes mellitus; T2D: type 2 diabetes mellitus; DR: diabetic retinopathy; Non-DR: nondiabetic retinopathy; *N*: number. Missing data: (a) 2; (b) 7.

**Table 3:** Health behavior of the study participants.

	T1D *N* = 52		T2D *N* = 334	
	DR *N* = 13 (%)	Non-DR *N* = 39 (%)	DR *N* = 112 (%)	Non-DR *N* = 222 (%)
Physical activity in the last month				
Every day/more times a week	6 (46.1)	26 (66.7)	61 (57.0)	118 (55.9)
Weekly	5 (38.5)	6 (15.4)	17 (15.9)	40 (19.0)
Only once in the last month/inactive	2 (15.4)	7 (17.9)	29 (27.1)	53 (25.1)^a^
Diet				
Yes	13 (100.0)	35 (92.1)	85 (77.3)	175 (81.8)
No	0 (0.0)	3 (7.9)	25 (22.7)	39 (18.2)^b^
Smoking				
Yes	5 (41.7)	6 (16.2)	8 (7.3)	21 (9.8)
Quit	2 (16.6)	8 (21.6)	38 (34.9)	74 (34.4)
Never	5 (41.7)	23 (62.2)	63 (57.8)	120 (55.8)^c^
Alcohol consumption				
Yes	7 (53.8)	11 (28.9)	35 (32.4)	79 (36.6)
No	6 (46.2)	27 (71.1)	73 (67.6)	137 (63.4)^d^

*P* < 0.05. T1D: type 1 diabetes mellitus; T2D: type 2 diabetes mellitus; DR: diabetic retinopathy; Non-DR: nondiabetic retinopathy; *N*: number. Missing data: (a) 16; (b) 11; (c) 13; (d) 11.

**Table 4:** Characteristics of the health status of the study participants.

	T1D *N* = 52		T2D *N* = 334	
	DR *N* = 13 (%)	Non-DR *N* = 39 (%)	DR *N* = 112 (%)	Non-DR *N* = 222 (%)
Hypertension	4 (30.8)	21 (55.3)	97 (87.4)	190 (88.4)
Systolic blood pressure (median, IQR, range)	153 (133-162)	135 (129-150)	130 (122-140)	130 (123-140)
	120-191	120-158	105-189	100-169
Diastolic blood pressure (mmHg) (median, IQR, range)	84 (80-85)	80 (70-85)	80 (75-85)	80 (70-85)
	78-95	58-90	60-104	60-101
Duration of hypertension (year) (median, IQR, range)	18 (3-42)	11 (7-20)	20 (10-40)	20 (10-37)
	3-52	2-53	2-56	3-56
Visual acuity				
<0.3	0 (0.0)	0 (0.0)	6 (16.7)	2 (5.5)
≥0.3	3 (100.0)	2 (100.0)	30 (83.3)	38 (95.0)^a^
HbA1c				
Elevated (≥7%)	13 (100.0)	37 (93.4)	88 (82.2)	170 (79.4)
Duration of diabetes (median, IQR, range)	20 (14-24)	20 (13-27)	13 (8-20)	15 (8-20)
	10-38	1-60	0-38	0-40
Diabetes medication	5 (41.7)	13 (34.2)	*86 (77.5)*	*187 (86.6)*
Diabetes in the family	6 (46.1)	21 (53.8)	52 (46.8)	124 (56.6)
Diabetic maculopathy	*7 (53.8)*	*2 (5.1)*	*81 (73.6)*	*15 (6.8)*

*P* < 0.05. T1D: type 1 diabetes mellitus; T2D: type 2 diabetes mellitus; DR: diabetic retinopathy; Non-DR: nondiabetic retinopathy; *N*: number; IQR: interquartile range. Missing data: (a) 305.

## Data Availability

Data from this study are available on request through the corresponding author.
